# RF Energy Harvesting Wireless Communications: RF Environment, Device Hardware and Practical Issues

**DOI:** 10.3390/s19133010

**Published:** 2019-07-08

**Authors:** Yu Luo, Lina Pu, Guodong Wang, Yanxiao Zhao

**Affiliations:** 1 Electrical and Computer Engineering, Mississippi State University, Mississippi State, MS 39759, USA; 2 Computer Science and Computer Engineering, University of Southern Mississippi, Hattiesburg, MS 39406, USA; 3 Computer Science, Massachusetts College of Liberal Arts, North Adams, MA 01247, USA; 4 Electrical and Computer Engineering, Virginia Commonwealth University, Richmond, VA 843068, USA

**Keywords:** RF Energy harvesting wireless communication (RF-EHWC), RF environment, RF-EHWC hardware, practical issues in RF-EHWC network design

## Abstract

Radio frequency (RF) based wireless power transfer provides an attractive solution to extend the lifetime of power-constrained wireless sensor networks. Through harvesting RF energy from surrounding environments or dedicated energy sources, low-power wireless devices can be self-sustaining and environment-friendly. These features make the RF energy harvesting wireless communication (RF-EHWC) technique attractive to a wide range of applications. The objective of this article is to investigate the latest research activities on the practical RF-EHWC design. The distribution of RF energy in the real environment, the hardware design of RF-EHWC devices and the practical issues in the implementation of RF-EHWC networks are discussed. At the end of this article, we introduce several interesting applications that exploit the RF-EHWC technology to provide smart healthcare services for animals, wirelessly charge the wearable devices, and implement 5G-assisted RF-EHWC.

## 1. Introduction

Radio frequency (RF) energy harvesting has been envisioned as a promising method to drive low-power wireless systems [1]. In addition to being a role as an information carrier, RF waves in wireless systems also work as an energy medium to power devices for environment sensing, wireless communications, and signal processing.

The RF energy is a perpetual resource that is widely present both indoors (e.g., Wi-Fi signal from wireless router) and outdoors (e.g., cellular and DTV signal). The RF energy harvesting wireless communication (RF-EHWC) technique introduces several attractive features into communication and sensing networks. For instance, it gives low-power wireless devices the self-sustainability, which allows those devices to operate semi-perpetually. In addition, by scavenging energy in the air, an RF-EHWC device can work without a chemical battery to achieve green communications. Moreover, RF-EHWC devices typically have light-weight structures compared to conventional facilities that collect renewable energy from wind, tide, and biomass. Therefore, they can be small in size, which allows them to work in applications where space and costs are critical [2,3].

The green, small-size, and sustainable features make the RF-EHWC technique suitable for a wide range of applications, such as the Internet of Things (IoT), body area networks (BANs), and smart infrastructures [4]. Those applications usually need the assistance of numerous wireless sensor nodes and low-power devices that have strong demands on sustainability and efficiency [5]. For instance, the wireless nodes deployed in the wilderness for environment monitoring and disaster warning should be green and durable. Obviously, RF-EHWC is a promising technique to meet the clean and self-sustainable requirements of these emerging applications.

### 1.1. Wireless Information and Power Transfer (WIPT)

The initial developments of wireless power transfer systems (WPT) and wireless information transfer (WIT) systems are independent. The former has a huge size in order to achieve efficient energy transfer at high-power levels [6], while the latter is often intended to build portable devices with low power consumption. Until 1948, Harry Stockman introduced a new concept, called the communication by means of reflected power [7]. His work is the prototype of the radio frequency identification (RFID), the first attempt to combine WIT with WPT.

Three decades later, a short-range RFID system was implemented based on the modulated backscatter [8]. In this system, the RF reader sent a 1 GHz continuous wave (CW) to an RF tag, which then changed the load on its rectifier to modulate the amplitude of backscatter waves for the data transmission. Since a portion of incident energy was rectified to power the RF tag for wireless communications, the passive RFID can be considered as the first WIPT system in the history. Nowadays, RFID and its derivatives, near-field communication (NFC) [9], have been widely used in different places for item classification, electronic payment and tool management [10,11].

**Figure 1:**
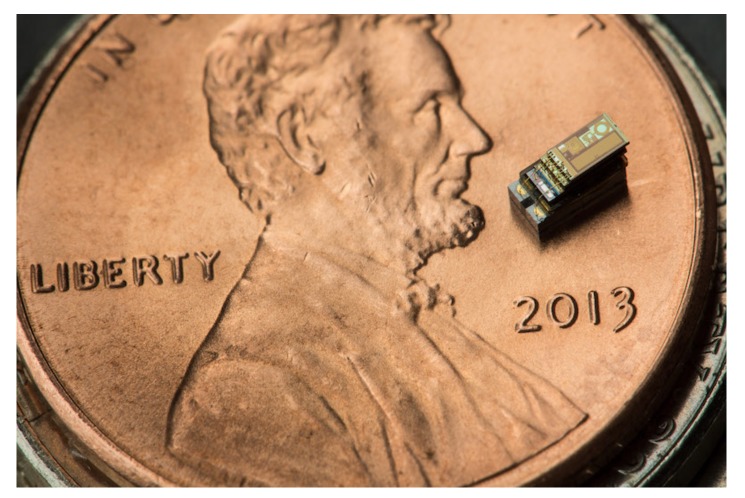
Smart dust developed by University of Michigan, sitting on a penny [16].

In 1990s, the concept of smart dust was proposed. As demonstrated in Figure 1, the smart dust is a miniature wireless mote that integratess multiple tiny microelectromechanical systems (MEMS) for environment sensing and wireless communications [12]. Different from the passive RFID that obtains energy from a dedicated RF reader, the smart dust can harvest energy from surrounding environment, such as solar, vibrations, or even ambient RF waves [13]. Owing to the features of miniaturization and sustainability, the smart dust is gradually penetrating into our daily life [14,15].

Simultaneous wireless information and power transfer (SWIPT) has caught considerable research attention for its efficient spectrum usage [17,18,19,20]. The SWIPT seeks to deliver wireless information and controllable energy concurrently in the same RF signal. Since information processing and energy harvesting cannot be simultaneously performed by the same circuit, the received signal has to be split into two distinct parts, one for energy harvesting and one for information decoding, in the time switching (TS) or power splitting (PS) manner. In the time switching (TS) structure, the RF signal is divided into two time slots, one for transferring power and the other for transmitting data. The power splitting (PS) structure splits the received signal into two streams, one with power ratio ρ for energy harvest and the other stream with power ratio 1−ρ for information decoding. By varying the length of data/energy slots in the TS scheme or adjusting the value of power ratio in the PS scheme, a balance can be achieved between the performance of information decoding and energy transfer. Extensive research has been conducted to investigate the rate-energy tradeoff for efficient design of SWIPT system [21].

### 1.2. Contributions of This Article

In SWIPT, the power transfer and information reception occur simultaneously. The receivers split the RF signal and use the collected energy to decode the information. Therefore, SWIPT is typically used for co-located energy/information access points (APs) to send data to one or more end users (i.e., downlink communications) [22]. In this article, however, we consider the scenario where the wireless devices harvest energy from ambient RF environment or dedicated energy sources and store the energy in a battery (e.g., supercapacitor). The harvested energy can be later utilized for uplink or ad hoc communications. Different from the simultaneous power transfer and data transmission in SWIPT, data transmissions in RF-EHWC take place after the devices have harvested a sufficient amount of energy.

Enabling energy harvesting brings new challenges for the design of wireless communication systems. First of all, both the energy harvesting efficiency and energy utilization are critical to RF-EHWC. This is because the RF energy suffers from a high spreading loss in the free space. As a result, the energy reaching the RF-EHWC device is very limited in a far-field RF environment [23]. Hence, the hardware (e.g., the energy harvesting circuit and the antenna) should be well designed to improve the energy harvesting efficiency; meanwhile, the software (e.g., the power management policy and the transmission strategy) should also be carefully developed to increase the efficiency of energy utilization. Second, in addition to collecting energy from the ambient RF, the RF-EHWC device can also actively request energy from associated base stations and access points in some applications. In this case, the interaction between the energy flow and the data flow is intricate since the energy transmission can interfere with the information decoding while the information transmission may interrupt the energy reception. Therefore, how to balance the power transmission and the communication throughput is a challenging issue in the RF-EHWC design [24]. Third, the energy harvesting capabilities add a new dimension to resource management in RF-EHWC networks. The new management policy not only needs to properly arrange data flows, but also needs to organize energy flows so that each RF-EHWC device can receive sufficient energy to power the system, and the network can also have a high throughput and a low power consumption [21].

Despite the growing interests from various research communities, there are still many unresolved problems to make the RF-EHWC realistic. In the past few years, most of research efforts have focused on hardware implementation and transmission management at the physical layer [25]. However, practical issues, such as the thin RF power density in the air, the nonlinear battery charging, intermittent connections in the RF-EHWC network, and security issues are still being overlooked. These under-explored topics inspire us to conduct more in-depth research on the development of practical RF-EHWC networks.

Following this direction, in this article, we survey the latest research progress achieved in the practical design of RF-EHWC over the past few years. The remainder of the article is organized as follows: In Section 2, we investigate the ambient RF environments and discuss the challenges on ambient energy harvesting. Section 3 introduces the hardware architecture of RF-EHWC systems and design principles of the RF-EHWC hardware circuit. Section 4 introduces the power management problem and discusses practical issues such as the nonlinear battery charging in an efficient power management design. A unique challenge, called the intermittent connection problem, to the resilient routing design in RF-EHWC networks is discussed in Section 5. The secure information transmission in RF-EHWC networks is presented in Section 6. Finally, we introduce several promising applications of the RF-EHWC technique in Section 7 and conclude this article in Section 8.

## 2. Urban and Semi-Urban RF Environment

The ambient RF energy is a perpetual resource to RF energy harvesting devices. In this section, we investigate the features of ambient RF energy and introduce the urban and semi-urban RF environments.

### 2.1. Power Restriction

**Figure 2:**
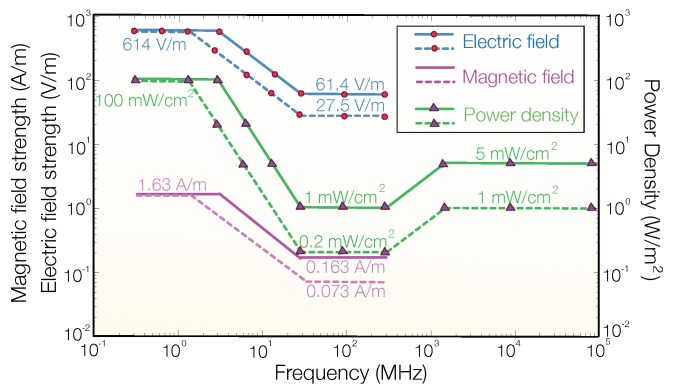
The limitation of MPE recommended by US FCC in 2013. The figure is created based on Table 1 in [27]. Solid lines and dot lines represent the MPE for controlled exposure ^†^ and uncontrolled exposure ^‡^, respectively. ^†^ Controlled exposure: Persons who are exposed as a consequence of their employment, provided those persons are fully aware of the potential for exposure and can exercise control over their exposure. ^‡^ Uncontrolled exposure: Members of the general public who are exposed as a consequence of their employment may not be fully aware of the potential for exposure or cannot exercise control over their exposure.

The higher frequency EM ionizing radiation, such as ultraviolet rays ($8\;\;\times \;\;10^{14}$ to $3\;\;\times \;\;10^{16}$ Hz), X-rays ($3\;\;\times \;\;10^{16}$ to $3\;\;\times \;\;10^{19}$ Hz), and gamma rays (>$10^{19}$ Hz), can break DNA molecules. For safety concern, the non-ionizing band that is between 30 kHz and 300 GHz is usually used for telecommunications [26]. Although the non-ionizing RF band is relatively safe to the body, an RF wave at sufficiently high power can burn tissues by converting energy into heat. To protect human from strong RF waves, there are several regulations on the maximal RF power density allowed in the air. According to the Federal Communications Commission (FCC) document published in 2013 [27], the advised maximal permissible exposure (MPE) of RF density to general public is 1 mW/cm${}^{2}$ on frequencies 1–100 GHz, as depicted in Figure 2.

In addition to the safety concern, the RF transmission power is further restricted to avoid interference for space reuse, especially in cellular and WiFi networks. As a consequence, the RF power density in most of the public area is much lower than the Maximum Permissible Exposure (MPE) limitation. Specifically, early results published in [28] show that in the very high frequency (VHF) and ultra high frequency (UHF) TV bands, the RF radiation power is lower than 0.1μW/cm${}^{2}$ for 95% population in US cities; only less than 1% population receive exposures higher than 1μW/cm${}^{2}$. The recent study tested in four US cities (Raleigh, NC; Spokane, WA; Wichita Falls, TX; and Colville, WA) also verifies that, in the common frequency bands, the strength of radio waves is below 1μW/cm${}^{2}$ over 90% area of the tested cities [29].

### 2.2. RF Distribution Features

According to measurement results, the RF power density in urban and semi-urban areas has two important features: (a) it has nonuniform spatial and spectrum distributions; and (b) its strength is relatively stable over time [23].

#### 2.2.1. Nonuniform Spatial Distribution

**Figure 3:**
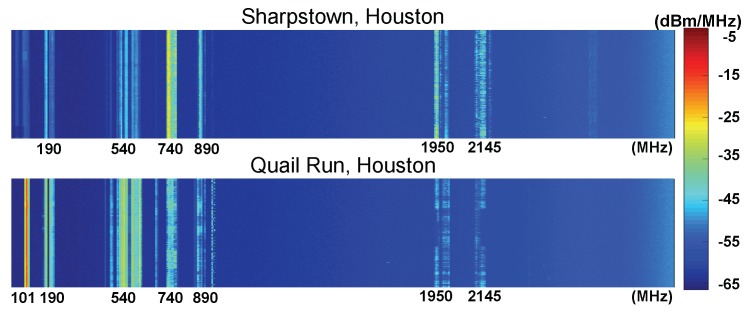
The RF power density measured at different places in Houston, TX.

**Figure 4:**
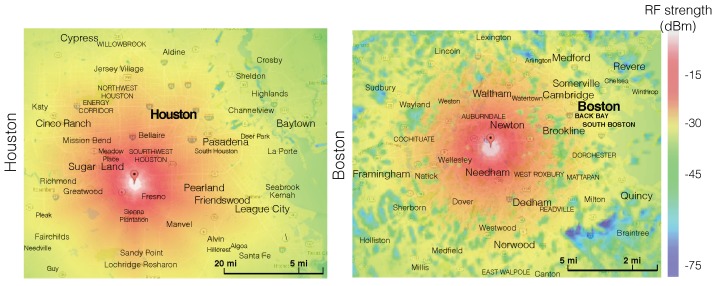
The influence of geography on the distribution of RF power density in Houston (plain area) and Boston (hilly area) drawn with TV Fool [33]. The effective radiated power (EPR) and the height above average terrain (HAAT) of TV station placed at Houston are 1 kW and 580 m, respectively [34]. The corresponding parameters of TV station located at Boston are 1.35 kW and 390 m, respectively [35].

**Figure 5:**
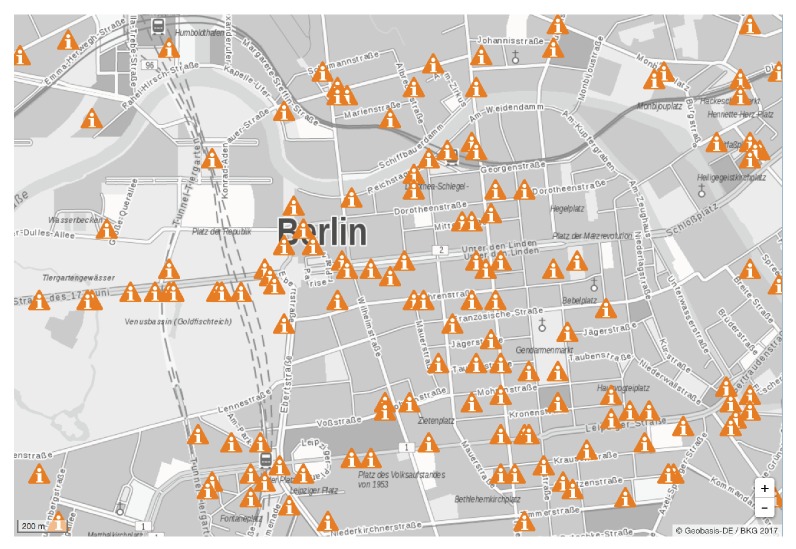
Nonuniform distribution of RF facilities around the center of Berlin, German [36].

The intensities of RF energy in different places have high heterogeneity due to the following factors:

- *High spreading loss*: As measured in [30], the total power density on 900 MHz and 1800 MHz can exceed 50 mW/m${}^{2}$ when the measurement site is 10 m from a cellular base station. However, this value quickly decreases to around 0.05 mW/m${}^{2}$ when measured 50 m away from the base station. The authors also measured the RF power density between 100 MHz and 3 GHz at different places in Houston and demonstrated the results in Figure 3. As shown in the figure, the KETH-TV signal on 540 MHz was around −30 dBm/MHz in Quail Run, Houston, which is about 1 mile away from the KETH-TV tower. In Sharpstown, Houston, which is about 10 miles away from the TV tower, the strength of RF signal on 540 MHz reduced to −50 dBm/MHz owning to the high spreading loss. In this situation, the TV signal on 540 MHz becomes too weak to be harvested by most of the RF-EHWC devices [31].
- *Block of obstacles*: Large obstacles can block an RF wave with a short wavelength. Mountains, slopes, and buildings that have a much larger size than the wavelength of radio signals can directly affect the spatial distribution of RF power density. As demonstrated in Figure 4, the signal attenuation is large but smooth around Houston (plain area); by contrast, it has dramatic fluctuations around Boston (hilly area). In addition to geography, buildings can also change the distribution of RF energy in the air. The results reported in [32] indicate that the power density in an indoor site is at least one order of magnitude lower than that in an outdoor one when two measurement sites have the same distance to a cellular base station.
- *Nonuniform deployment of RF facilities*: The number of RF facilities deployed in an area is usually related to the population density. Consequently, the nonuniform distribution of population causes a nonuniform deployment of RF facilities, which results in the nonuniformity of RF power density in the environment. In Figure 5, we show the distribution of RF facilities in Berlin, which is published by the German Federal Network Agency in 2017 [36]. Figure 3 also demonstrates the heterogeneity of spectrum distribution resulted from the nonuniform deployment of RF facilities. Since the Klol-FM and KETH-TV stations are built near Quail Run, Houston, we can observe strong RF strength on 101 MHz and 540 MHz bands in that area. In the downtown and midtown areas such as Sharpstown, Houston, the strength of FM and TV signals becomes negligible while the Global System for Mobile Communications (GSM) and Long-Term Evolution (LTE) cellular bands (i.e., 740 MHz and 890 MHz in the top graph of Figure 3) are significant.

#### 2.2.2. Nonuniform Spectrum Distribution

Due to the broadcast nature of wireless communications, RF signals radiated by different telecommunication systems coexist in the air. The strength of signals from different RF sources can have a big difference, as listed in Table 1. According to the measurements for ambient RF power in Boston [23], the highest power density (84 nW/cm${}^{2}$) that appears around 1800 MHz is three orders of magnitude higher than the power density (0.18 nW/cm${}^{2}$) measured around 2.4 GHz.

The difference of RF power density on the spectrum is mainly caused by the huge difference in the transmission power and deployment density of different RF facilities. For instance, the transmission power of a cellular microcell base station is usually from 1 W to 5 W [30], which can cover only hundreds of meters. Hence, the deployment density of microcell stations is usually high to provide a reliable mobile communication service. As a contrast, the transmission power of a TV tower can be as high as 100 kW on the VHF band or 5 MW on the UHF band in order to cover users within 97 km [41]. Therefore, the distribution of TV towers can be sparse.

Due to the nonuniform distribution of RF power density on the spectrum, an RF-EHWC device needs to select a proper frequency band to harvest energy. For instance, in the center of a city, it may be far from a TV tower but surrounded by multiple microcell mobile base stations. In this case, harvesting energy in the cellular band is a wise choice. By contrast, if an RF-EHWC device is closer to a TV, it will be better to harvest energy in the TV band.

#### 2.2.3. Temporal Features

**Figure 6:**
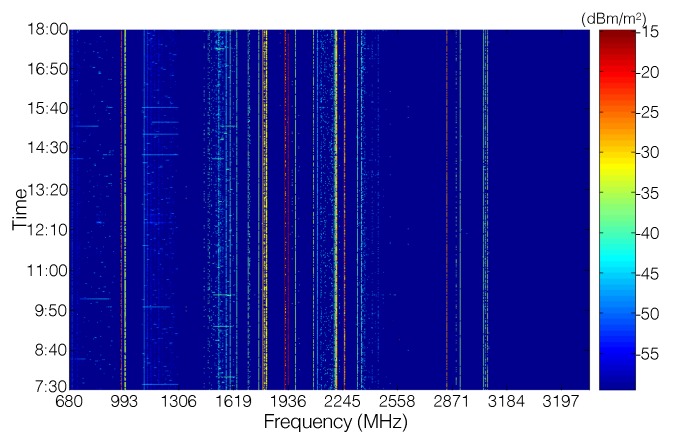
RF power density in an out-door environment with respect to time and frequency [42].

Although the power density of ambient RF signal varies with locations and frequencies, it is relatively stable in the time-domain; especially in an outdoor environment, there are only small fluctuations in the RF power density over time. This can be observed in Figure 6, where the power density measured in a certain frequency forms a monochromatic line over time. According to the data published in [42], in an urban area, the total power density in the 680 MHz–3.5 GHz frequency band just has around 3 dBm/m${}^{2}$ fluctuation in the daytime. (In an indoor environment, the RF signal concentrates in the frequency band of a wireless local area network (WLAN). According to the data collected by [43,44], the strength of RF signals is quite stable during the late night and early morning, but varies a lot in the daytime due to human activities. In addition, as reported in [43], the average intensity of receiving signals measured in the daytime is about 8 dBm higher than that in the night and morning.)

### 2.3. Weather Dependent Attenuation

**Figure 7:**
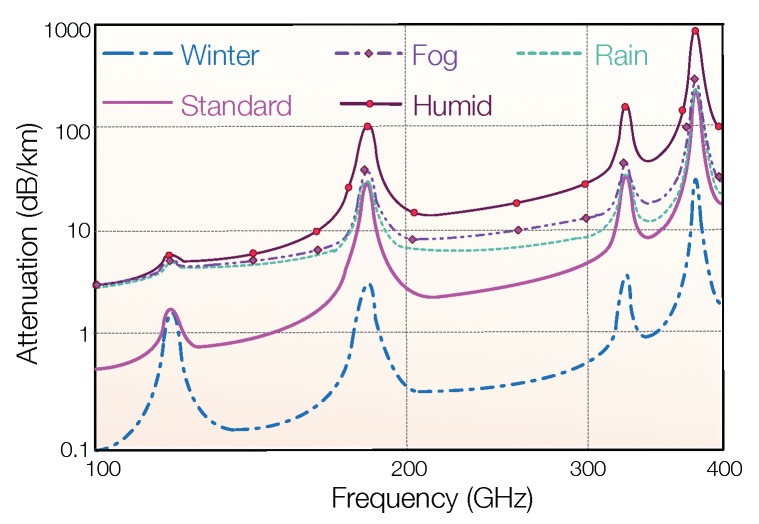
Propagation attenuation of electromagnetic waves at the sea level in different weathers (recreated from Figure 2 in [45]).

In the outdoor environment, the weather can considerably impact the propagation attenuation of EM waves. As shown in Figure 7, the attenuation of EM waves in sunny winter is as low as 0.1 dB/km. If the weather is fog, rain, or the air is humid, the attenuation increases to 4 dB/km, 40 times higher than that on a sunny day. For an energy harvesting communication system, the variation of propagation attenuation not only impacts the communication range but also affects the energy harvesting rate. Therefore, to improve the reliability of an RF-EHWC system in different weather conditions, a system designer needs to reserve sufficient margin for the power consumption and receiving SNR when making a communication budget.

Understanding the temporal and spatial features of ambient RF power density is essential to the efficient hardware design, power management, as well as routing and security design. The energy harvester is designed to work in a specific narrowband signal. As discussed in Section 3, the power conversion efficiency of the energy harvesting circuit severely depends on the strength of the RF signal. Therefore, the design of energy harvester needs to know both the central frequency and the range of signal strength for the highest power conversion efficiency. In Section 4, we introduce that efficient power management methods need to know the amount of energy that can be harvested in the near future, which relies on the temporal feature of the RF signal strength. In Section 5 and Section 6, we also discuss how the low power density of ambient RF signal challenges the routing and security design in the RF-EHWC networks.

## 3. RF-EHWC Hardware

An RF-EHWC device combines the RF energy harvest and wireless information transmission techniques. To better understand the working principle of the RF-EHWC device, we briefly introduce the hardware architectures of RF-EHWC devices. Depending on whether the energy harvesting circuit and the communication module share the antenna, RF-EHWC systems can be divided into the separate system or co-located system.

**Figure 8:**
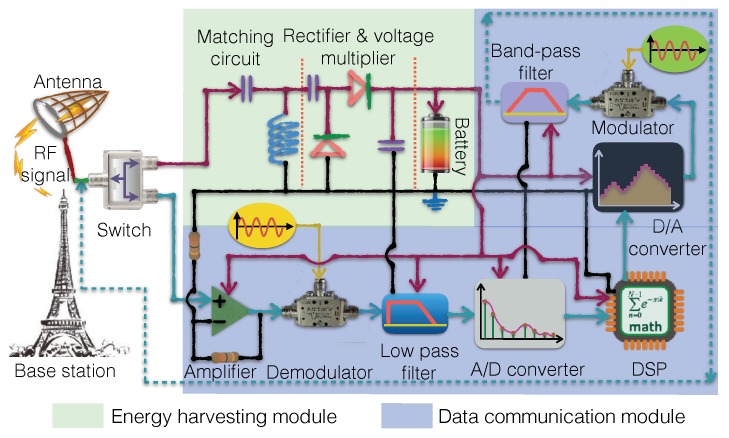
An RF-EHWC system, where the red lines indicate the energy flow of the energy harvester charging the components of the communication module.

In a separated system, the energy harvester has a dedicated antenna and therefore can be considered as a battery, which replenishes energy automatically and supplies power to the wireless communication module. In a co-located system, the energy harvester and the communication module share the same antenna, as shown in Figure 8. It switches between energy harvesting and information communication modes in a time-division manner. Sharing an antenna between the energy harvester and the communication module can reduce the system size of a co-located system. However, it usually requires the energy stream and the information stream to have the same frequency band. Meanwhile, it will incur additional requirement on the RF-EHWC hardware design, as introduced below.

### 3.1. Design Principles of RF Energy Harvester

The energy harvester of an RF-EHWC system consists of three key components: an impedance matching circuit, a rectifier, and a voltage multiplier, as shown in Figure 8. Next, we discuss the design principle of each component of energy harvester.

#### 3.1.1. Impedance Matching Circuit

To maximize the efficiency of energy conversion, the input impedance of a receiving antenna and the output impedance of the receiving circuit should be identical; otherwise, part of radio energy received by the antenna will be reflected back to the environment.

The matching circuits for energy harvest and data reception are slightly different. As illustrated in Figure 8, for the data reception, the antenna’s load is a front-end amplifier, which usually has 50Ω input resistance. Therefore, the antenna can simply match its output impedance to 50Ω through a transmission line, which is typically a coax cable. For the energy harvest, the antenna’s load is a rectifier and a voltage multiplier. Its input impedance depends on the structure of the circuit. Accordingly, we need a specifically designed matching circuit between the antenna and the rectifier to match the input impedance of the rectifier with the output impedance of the receiving antenna [3]. The matching circuit is composed of inductive and capacitive components, as shown in Figure 8.

#### 3.1.2. Rectifier and Voltage Multiplier

**Figure 9:**
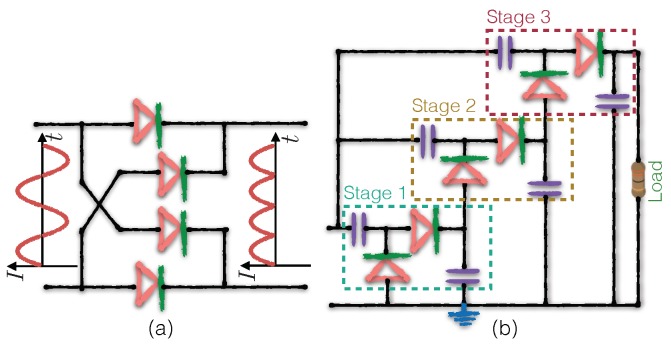
(**a**) Graetz bridge rectifier; and (**b**) three-stage Dickson voltage multiplier.

The alternating current (AC) output of a matching circuit cannot be directly used to charge an energy storage component (i.e., battery). The purpose of rectifier is to transform energy from AC to direct current (DC). The basic rectifier circuit consists of a few diodes to rectify the output to DC. Figure 9a shows a typical full-wave Graetz bridge rectifier.

Due to the low power density of RF signal in the air, the output voltage of the rectifier may not be high enough to charge a battery or to drive a wireless communication module. In this situation, a voltage multiplier is implemented to boost the output voltage of the rectifier. In a real RF energy harvest circuit, the rectifier and voltage multiplier are usually implemented together in cascaded subnetworks consisting of capacitors and diodes [46], as shown in Figure 9b. In the figure, each subnetwork is referred to as a “stage”; each stage marks the output of the previous stage as a biasing reference. Using this approach, the subnetworks can pump up the output voltage stage-by-stage to reach the desired voltage.

Schottky diodes and PN diodes are two options to build the rectifier and voltage multiplier. The main advantages of the Schottky diode are the low forward voltage (the forward voltage is the voltage drop across a diode if the voltage at the diode’s anode is more positive than the voltage at its cathode) and small junction capacitance [47]. The Schottky diode can offer as small as 150 mV of forward voltage and 0.2 pF of junction capacitance, much lower than 700 mV of forward voltage and few picofarads of junction capacitance in a typical PN diode. For an RF energy harvester, the low forward voltage of the Schottky diode can improve the efficiency of power conversion, while the low junction capacitance can increase the maximum frequency of RF energy that a system can harvest.

Despite its advantages, the Schottky diode is usually produced as discrete components and, therefore, integrating the Schottky diode into a standard CMOS process will significantly increase the fabricating costs. By contrast, the PN diode based rectifier and voltage multiplier can be incorporated into a single chip, which has apparent advantages on high consistency and low cost. Those features make the PN diode attractive to RF-EHWC systems that have high demands on the portable size, low cost, and high reliability.

### 3.2. Concerns in the Design of Energy Harvesting Circuits

The performance of the energy harvesting module is usually evaluated in the following aspects:

- *Power conversion efficiency* is defined as the ratio of the DC output power to the incident RF power. It characterizes the ability of energy harvester to convert RF energy to DC power and is mainly determined by the structure of the energy harvesting circuit, the frequency and the strength of incident waves.
- *Energy sensitivity* is the minimum incident power to activate the energy harvester. It is affected by the voltage gain of the voltage multiplier. Energy sensitivity is an important metric to guarantee that an RF-EHWC system can work reliably in an RF environment with a low power density.

**Figure 10:**
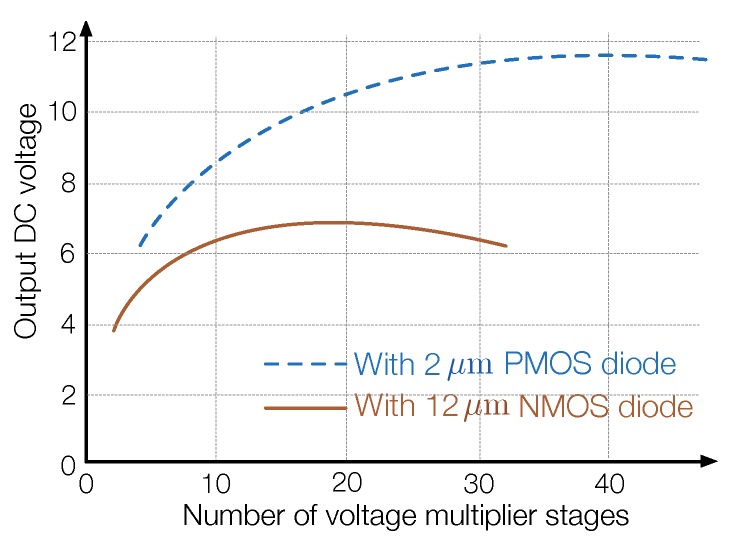
Output DC voltage with respect to the number of stages in a voltage multiplier (recreated from Figure 9b in [48]).

It is worth noting that there is a tradeoff between energy sensitivity and power conversion efficiency, as illustrated in Figure 10 [3]. Specifically, although a voltage multiplier with more stages can provide higher voltage gain, the parasitic capacitance in the circuit also increases linearly, which reduces the power conversion efficiency of the antenna, thereby decreasing the input voltage at the initial stage of the multiplier. Therefore, the number of stages in the voltage multiplier needs to be carefully determined based on the incident power of RF signal to maximize the amount of energy that can be harvested.

**Figure 11:**
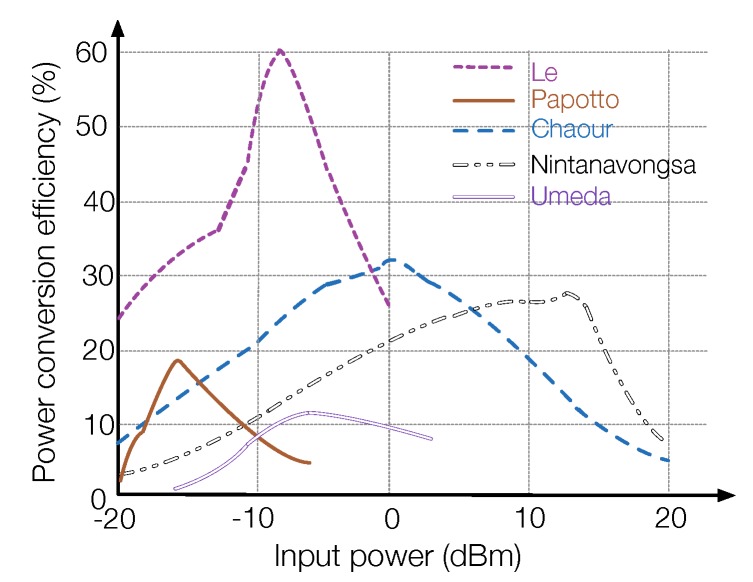
Power conversion efficiency of the circuits designed by Le [48], Papotto [50], Chaour [49], Nintanavongsa [3], and Umeda [51].

Another important feature is that the power conversion efficiency of the energy harvester does not monotonically increase with the intensity of the incident power, due to the nonlinearity of diodes and the parasitic influence of the used elements [49]. When the incident energy is very strong, the power conversion efficiency will have a sharp decrease as the voltage swing at the diode exceeds the breakdown voltage. In this case, the diode conducts in the reverse direction. The nonlinear feature of varied energy harvesting circuits is presented in Figure 11. Due to the high spreading loss of Electromagnetic waves in the free space, the RF-EHWC devices may receive RF signals of strongly varying incident power at different locations. In this case, the breakdown effect needs to be considered carefully.

Take a real RF-EHWC system, Powercast P2110-EVB (from Powercast, PA, USA), as an example. The Powercast P2110-EVB achieves the highest power conversion efficiency at 900 MHz [52]. Assume the power of the radiated signal from a dedicated energy source is 60 W (47.8 dBm) [26], then the incident power is only 4.2μW (−23.8 dBm) at 100 m, which lies in the low power region in Figure 11. However, when the Powercast P2110-EVB is close to a mobile phone, which is an energy source providing 250 mW (the maximum power that mobile phones are permitted to transmit at 900 MHz frequency band is 2 W, but the average power transmitted by a phone is commonly lower than one-eighth of this maximum value, i.e., 0.25 W [26]), then the strength of the incident RF power can reach 7.9 mW (9 dBm) at 15 cm distance. According to Figure 11, this input power at this level is high enough to trigger the breakdown effect in the diode.

**Figure 12:**
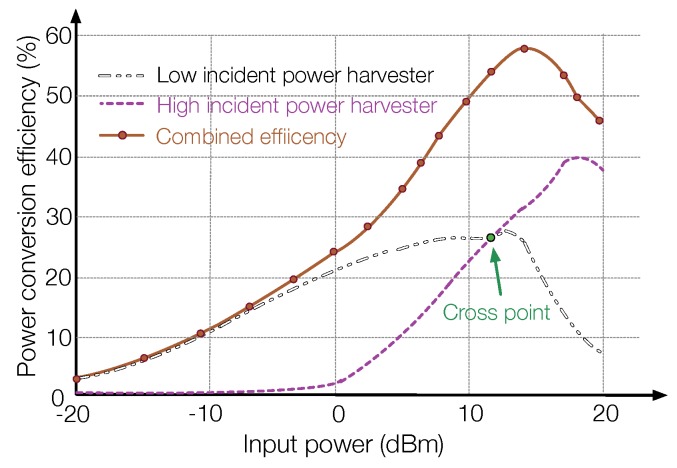
Combined power conversion efficiency of two different energy harvesters (recreated from Figure 20 in [3]).

To make an RF-EHWC system work efficiently in a high dynamic RF environment, a feasible solution is to use multiple complementary energy harvesting modules. In [3], a dual-stage energy harvesting circuit is introduced, where two separate energy harvesting modules are optimized for different input power. The first harvester has an HSMS-2822 diode based seven-stages voltage multiplier that has a low forward voltage (150 mV) but a small breakdown voltage (2 V) to achieve high efficiency at the low input power. The second harvester uses the HSMS-2822 diode that has a high breakdown voltage (15 V) but a large forward voltage (340 mV), which works efficiently at a high power density. As shown in Figure 12, through carefully arranging the crossover region of multiple energy harvester, the RF-EHWC system can efficiently harvest energy with a wide range of incident power.

## 4. Power Management in RF-EHWC

Considering the low density of the RF signal, it is crucial to efficiently utilize the harvested energy of limited amount. Many power management strategies have been proposed in recent years. Those strategies dynamically adjust the transmission power of wireless communications based on the energy harvesting rate and traffic load. As discussed in the following subsections, an appropriately designed power management policy can significantly improve the throughput and reduce the transmission latency of an RF-EHWC device.

### 4.1. Design Principles of Power Management in RF-EHWC

#### 4.1.1. Limited Energy Resource

**Figure 13:**
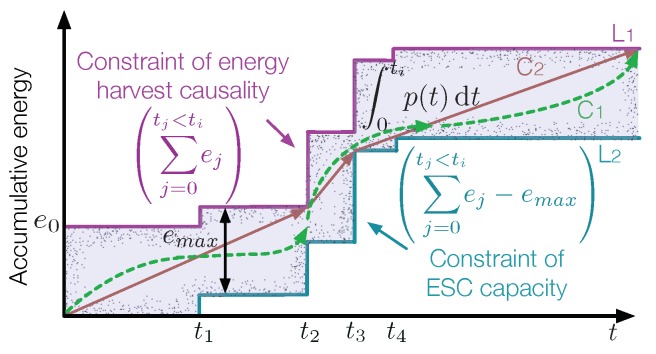
Power management with limited energy resource.

To facilitate the description of an energy harvesting process, RF waves arrived at an RF-EHWC system are generally modeled as discrete energy packets with varying sizes and random time of arrivals. In conventional offline power management approaches, it is usually assumed that the time and the number of data packets and energy packets that will arrive in the future are known. The accumulative energy harvested by the system is a staircase curve, which is represented by L${}_{1}$ in Figure 13. Due to the limited capacity of its battery, an additional staircase curve L${}_{2}$ is obtained by shifting L${}_{1}$ down by $e_{max}$, representing the capacity constraint of the battery. The profile of accumulative energy consumption, P, should stay inside the feasible energy tunnel: if it exceeds the upper bound, L${}_{1}$, the battery will not be able to provide sufficient energy; if the curve P is below L${}_{2}$, the energy will overflow from the battery. The optimal transmission scheduling is thus formulated as the following problem to find the consumption curve P that maximizes the data throughput given the limited energy resource.

(1) $$\begin{array}{cc} & P1:\;\;\;\;\underset{r(t)}{\arg\;\max}\int_{0}^{t_{i}}r\left(t\right)\;dt,\\ s.t. & C1:\;\;\;\;\int_{0}^{t_{i}}p\left(t\right)\;dt\leq \sum_{j=0}^{t_{j}\leq t_{i}}e_{j},\;\;\;\;\;\;\;i=1,\ldots ,N+1,\\ & C2:\;\;\;\;\sum_{j=0}^{t_{j}\leq t_{i}}e_{j}-\;\int_{0}^{t_{i}}p\left(t\right)\;dt\;\leq e_{max},\;\;\;i=1,\ldots ,N, \end{array}$$

where $e_{i}$ and $t_{i}$ are the amount of energy and the arrival time of energy packet *i*, respectively; $e_{max}$ is the maximum capacity of the battery; and p(t) is the instant transmission power of the communication module at time *t*, which is related to the transmission power through a power-rate function, p(t)=f(r(t)). Constrains ***C*1** and ***C*2** represent the energy causality constraint and the maximal battery capacity constraint, respectively.

The work presented in [53] proves that the optimal transmission power of an RF-EHWC device should remain unchanged between energy harvests in order to maximize the throughput. With such a conclusion, power management is simplified into a piecewise-linear optimization problem. It is pointed out that the optimal transmission power in the feasible energy tunnel has the following properties [54]:

**Property** **1.** *The optimal transmission power does not change until the battery is either full or completely depleted.*

**Property** **2.** *The optimal transmission power decreases only at energy arrival instants when the battery is full and increases only at energy arrival instants when the battery is depleted.*

**Property** **3.** *The optimal power management keeps the longest stretches of constant periods in the feasible energy tunnel.*

In addition to the solution from a graphical point of view, a directional water-filling algorithm is proposed in [55], where the energy is considered as “water” that has two features: (a) it cannot flow back due to the constraint of energy causality; and (b) it can be stored for future use, but the amount of water flowing to the future each time cannot exceed the maximum capacity of the battery. To maximize the throughput, the directional water-filling algorithm needs to distribute the water as equal as possible along the timeline.

#### 4.1.2. Limited Data Storage

Due to the cost and the size constraints, the maximum capacity of the data storage component (i.e., data buffer) in an RF-EHWC system is limited. Therefore, the collected data may overflow from the data buffer if they cannot be sent out timely. The optimal power management with the consideration of limited data storage is to seek an energy harvesting strategy to transmit the data timely at the minimum energy cost. A dedicated energy source is usually needed to charge the RF-EHWC device in a fully controlled manner. In [56], the authors developed a dual-tunnel strategy to solve this problem.

In Step (a) of the dual-tunnel strategy, it calculates the least energy that the RF-EHWC system needs to send the collected data subject to the limited capacity of data storage. We can formulate the objective of the first step as the following optimization problem:

(2) $$\begin{array}{cc} & P2:\;\underset{p(t)}{\arg\;\min}\int_{0}^{t_{i}}p\left(t\right)\;dt,\\ s.t. & C1:\;\;\int_{0}^{t_{i}}r\left(t\right)\;dt\;\leq \;\sum_{j=1}^{t_{j}\leq t_{i}}\;\;D_{j},\;\;\;\;\;\;\;i=1,\ldots ,N+1,\\ & C2:\;\;\sum_{j=1}^{t_{j}\leq t_{i}}D_{j}-\;\int_{0}^{t_{i}}r\left(t\right)\;dt\;\leq \;D_{max},\;\;\;\;i=1,\ldots ,N+1, \end{array}$$

where $D_{i}$ and $t_{i}$ are the size and the arrival time of data packet i, respectively; $D_{max}$ is the maximum capacity of the data storage; and the transmission rate is represented by r(t), which is related to the transmission power through a power-rate function, p(t)=f(r(t)). Constrains ***C*1** and ***C*2** represent the data causality constraint and the maximal data capacity constraint, respectively.

**Figure 14:**
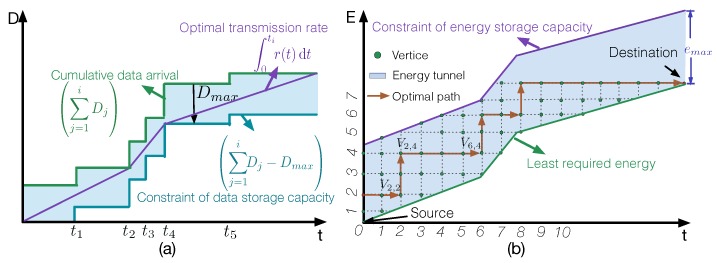
Step (**a**): Data transmission scheduling under the constraint of limited data storage, where $D_{j}$ and $t_{j}$ are the amount of data and the arrival time of data packet *k*, respectively; $D_{max}$ is the maximum capacity of the data storage; and r(t) is the instant transmission rate of an RF-EHWC device at time *t*. Step (**b**): Schedule the energy request to minimize the energy cost subject to the least required energy obtained in Step (**a**). Here, vertical edges indicate energy harvests and incurs overhead for energy request.

Here, Problem **P2** is to minimize the energy consumption of the EHD on transmitting all data, subject to the limited data storage. As shown in Figure 14a, a feasible data tunnel is constructed based on the cumulative data arrival and constraint of data storage capacity. By comparing Problems **P2** and **P1**, it can be realized that managing the transmission power in a feasible data tunnel is similar to the optimization problem introduced in Section 4.1.1. Thus, the optimal transmission power in a feasible data tunnel must have the following three properties:

**Property** **4.** *The optimal transmission power does not change until the data buffer is either full or empty.*

**Property** **5.** *The optimal transmission power decreases if the data buffer is completely filled or increases if the storage is empty.*

**Property** **6.** *The optimal power management keeps the longest stretches of constant power periods in the feasible data tunnel.*

The optimal transmission power solved in Step (a) forms an energy tunnel, as shown in Figure 14b. The lower bound is the least required energy calculated in the first step. The width of the tunnel is the maximum capacity of the battery. If the profile of cumulatively requested energy (the red line in Figure 14) is below the lower bound, the harvested energy will be insufficient for a timely data transmission and will cause data overflow; if it is over the upper bound, the requested energy will be larger than the battery’s capacity and will result in energy overflow.

In Step (b) of the dual-tunnel strategy, it seeks an energy request strategy within the feasible energy tunnel to minimize the energy cost at the energy source. A path-oriented approach is proposed in [57] for the optimal energy request. The new feasible energy tunnel is divided into multiple grids, forming a graph with a set of “vertices” and “edges”, as depicted in Figure 14. A horizontal edge means no energy charging and generates no cost at the energy source. The vertical edge indicates an energy replenishment, which produces an associated cost for the energy request. Eventually, the optimal energy requesting strategy in the path-oriented approach is converted to finding a route from the source to the destination that has the minimum sum-cost along the path in a directed and weighted graph. Dynamic programming methods can be applied to solve the converted problem.

#### 4.1.3. Packet Deadline Constraint

In some delay-sensitive applications, such as target detection and disaster warning, the sensing information must be delivered before a transmission deadline. How to minimize energy consumption while sending messages timely involves the third category of power management problem in RF-EHWC.

**Figure 15:**
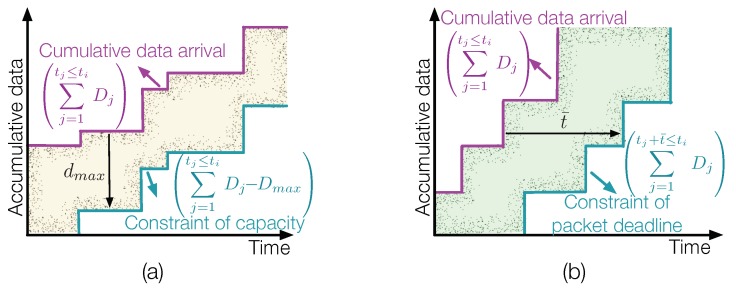
(**a**) A feasible data tunnel with the limited data storage, where $d_{max}$ is the maximal capacity of data storage. (**b**) A feasible latency tunnel with the packet deadline constraint, where $\bar{t}$ is the maximal latency allowed for each data transmission.

Similar to the feasible data tunnel built in Figure 15a, we can build a feasible latency tunnel, where the upper bound is the cumulative data arrival. The lower bound of the tunnel is determined by the transmission deadline of data packets. As investigated in [58], if all data packets have the same deadline and the transmission follow an earliest-deadline-first service, then the lower bound of the tunnel can be obtained by simply shift the upper bound right by the timeline, $\bar{t}$, as illustrated in Figure 15b.

(3) $$\begin{array}{cc} \; & P3:\;\underset{p(t)}{\arg\;\min}\int_{0}^{t_{i}}p\left(t\right)\;dt,\\ \;s.t.\; & C:\;\;\sum_{j=1}^{t_{j}+\bar{t}\leq t_{i}}D_{j}\leq \int_{0}^{t_{i}}r\left(t\right)\;dt\;\leq \sum_{j=1}^{t_{j}\leq t_{i}}D_{j},\;\;\;\;\;\;\;i=1,\ldots ,N+1, \end{array}$$

where $D_{i}$ and $t_{i}$ are the size and the arrival time of data packet i, respectively; $\bar{t}$ is the packet deadline constraint; and the transmission rate of the EHD r(t) and the transmission power p(t) are correlated through a power-rate function, p(t)=f(r(t)). The right half of the constraint **C** represents the data causality constraint and the left half indicates the constraint of packet deadline.

In a more general case, an RF-EHWC system can generate data packets having different priorities and transmission deadlines. In this situation, $\bar{t}$ in Figure 15b becomes a variable. The results obtained in [58] show that if we ignore the energy constraint, i.e., assuming an RF-EHWC device always has sufficient energy for transmitting the data at required rates, the optimal power management with dynamic delay constraint on each data packet is quite similar to the optimal power management with limited data storage obtained at Step (a) in Section 4.1.2. In other words, the profile of the cumulative data sent by the RF-EHWC device is graphically the tightest string in the feasible latency tunnel with a varying $\bar{t}$.

### 4.2. Practical Issues in Power Management

The aforementioned power management just considers the limited battery capacity, limited data storage capacity or constrained packet deadline, respectively. In this section, we introduce some real features of the energy harvesting circuits and their impact on the realistic power management in RF-EHWCs.

#### 4.2.1. Nonlinear Battery Charging

**Figure 16:**
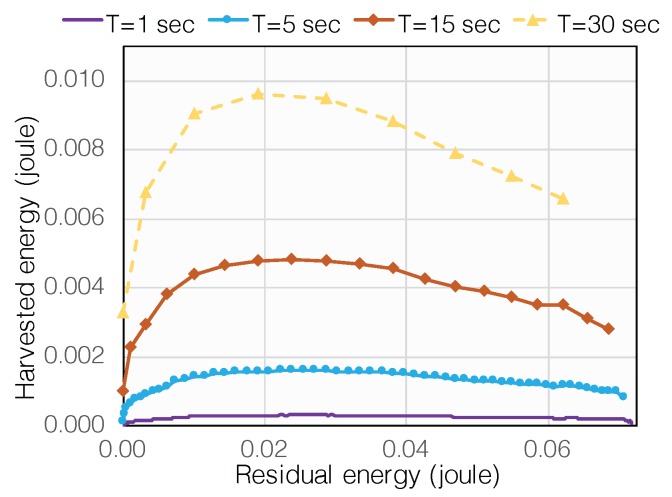
Nonlinear energy harvesting process with respect to the residual energy, where *T* is the length of the energy packet.

The nonlinear charge is a well-known characteristic of batteries (e.g., supercapacitor, Lithium-ion battery, etc.) [59]. The amount of energy harvested from an energy packet not only depends on the power density and the duration of energy packets but also on the residual energy in the battery. As verified in the experiment reported in [60], when the power and length of energy packets are fixed, the amount of harvested energy in each energy replenishment is a concave and non-monotonic function of the residual energy in the battery, as demonstrated in Figure 16. The nonlinear relation between the harvested energy and the residual energy in the battery is raised in [61] and formulated in [60].

**Figure 17:**
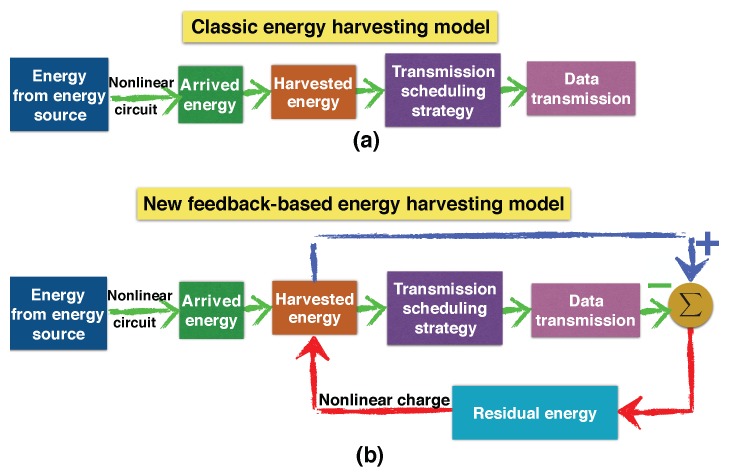
(**a**) Classic energy harvesting model without considering the nonlinear charging characteristic. (**b**) New feedback model with the nonlinear battery charging considered [60].

In the classic energy harvesting, the harvested energy is modeled as a random but predetermined sequence, as shown in Figure 17a. The existing work in optimal transmission scheduling strategy is to seek a curve within such a fixed energy tunnel so that a pre-defined objective is optimized (e.g., maximizing throughput) [54,55]. Unfortunately, the above assumption is not true in real energy harvesting systems. Since residual energy is affected by the data transmission, the amount of energy that will be harvested from an energy packet is not a predetermined value but a variable that heavily depends on the data transmission strategy. In other words, data transmission influences harvested energy in a real-time fashion; an RF-EHWC device cannot estimate what amount of energy it can harvest before scheduling its transmissions in the offline optimal transmission scheduling.

To accurately describe the energy harvesting process, a new model is proposed in [60], as illustrated in Figure 17b. In the new model, an energy feedback loop from the data transmission strategy to the harvested energy is formed, which describes the impact of nonlinear charging on the energy harvesting process. The nonlinear battery charging makes optimal power management become a challenging problem. On the one hand, for the purpose of throughput maximization, an efficient offline power management needs to manage the transmission power based on the amount of energy that can be harvested in the future; on the other hand, the power management strategy itself can affect the energy harvesting process through adjusting the residual energy in the battery.

To address the above problem, we can apply a recursive algorithm derived from the Karush–Kuhn–Tucker (KKT) conditions. (The KKT approach, allowing inequality constraints, generalizes the Lagrange multipliers method, which allows only equality constraints. KKT conditions are necessary conditions. Therefore, the solutions to the KKT approach need to be further verified if they are sufficient for optimality. The KKT approach has been used in solving nonlinear programming problems in wide areas [62,63].) As studied in [60], assume a total number of N energy packets arrive at an RF-EHWC device, and let $E_{i}^{r}$ be the residual energy at the moment of energy packet i arrival. By leveraging the inherent relationship among the residual energy, the transmission power, and the harvested energy, $E_{i}^{r}$ can be represented by $E_{1}^{r}$ through the energy harvesting function, i.e., $E_{i}^{r}=Q_{i}\left(E_{1}^{r}\right)$, where i=2,…,N. Afterwards, according to the principle that the RF-EHWC system must use all harvested energy at the end of data transmission to maximize the throughput, it can be obtained that $Q_{N+1}\left(E_{1}^{r}\right)\;=\;0$. By solving this high order nonlinear equation, $E_{1}^{r}$ is obtained. Eventually, substituting $E_{1}^{r}$ into $Q_{i}$, we can calculate the optimal amount of energy that should be retained in the battery when energy packets arrive. Thereafter, the RF-EHWC system can manage its transmission power efficiently based on the optimal residual energy.

#### 4.2.2. Battery Imperfection

Battery imperfections include energy leakage, capacity degradation over time and imperfect knowledge of battery status. Next, we investigate the impact of battery imperfection on power management.

The energy stored in a battery always gradually leak with time due to the off current in a circuit and the self-discharge characteristic of the battery [64]. Depending on the material used in a battery, the self-discharging rate can range from 2% to 50% per month (e.g., Lithium-ion: 2%; Nickel-cadmium: 15%; NiMH: 30%; and supercapacitor: 50%) [65,66]. The impact of energy leakage on power management can be solved by alternatively interpreting the leadage as the operation power of the circuit [64], which is discussed in Section 4.2.3.

**Figure 18:**
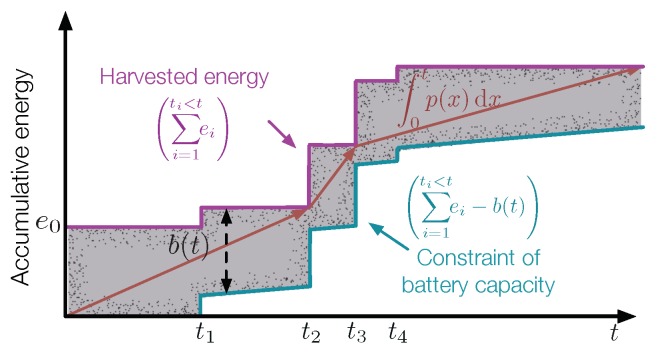
Power management with the time-varying battery capacity, where b(t) is the time-varying battery capacity. $e_{i}$ and $t_{i}$ are the amount of harvested energy and the arrival time of energy packet i, respectively. p(t) is the instant transmission power of an RF-EHWC device at time *t*.

The capacity degradation of the battery modifies the shape of feasible energy tunnel, i.e., the tunnel width monotonously decreases reflecting the time-varying battery capacity, as shown in Figure 18. According to the piecewise linear feature of the optimal transmission strategy, the transmission power changes only when new energy is harvested. This implies that the time-varying battery capacity that modifies the tunnel width will not affect the optimal power management in between two adjacent energy replenishment. Therefore, b(t) can be modeled as discrete values, $b_{t_{i}}$, where $t_{i}$ is the time of the ith energy harvest [64]. Conventional solutions to piecewise linear optimization problem can be used to solve the optimal power management with the consideration of battery capacity degradation.

Another type of battery imperfection comes from the imperfect knowledge of the instant battery level (e.g., the node may only know if the battery is low or high). Most of power management policies introduced in Section 4 may fail to manage the transmission power efficiently without the exact knowledge of the battery state. The authors of [67] investigated the features of limited knowledge of battery level and proposed a partially observable Markov decision process (POMDP) model. Based on the historical observation of energy harvest in the past, it maintains a probability distribution of the residual energy over a set of possible states. To maximize a reward, which is the long-term throughput, the system makes the best decision on the data transmission based on the limited knowledge.

#### 4.2.3. Circuit Power

In the non-ideal circuit, its consumed power is the sum of transmission power and circuit power, which includes the energy dissipated by the microprocessor, AD/DC converter, amplifier, and filter. The non-negligible circuit overhead affects the profile of energy consumption, $\int_{0}^{t}p\left(x\right)$. As a consequence, the energy-rate function is no longer monotonic but first increases and then decreases with the transmission rate [68].

It is verified that the continuous transmission becomes inefficient and on–off transmission is advocated as an alternative [69,70,71]. As proposed in [69], if many packets are reaching their transmission deadline, the system transmits with a high power to catch the deadline. In other cases, the device sends the data with the rate $r_{m}$ to maximize energy efficiency. Once a transmission is completed, the system enters a sleep mode immediately. A two-phase transmission scheduling policy is provided in [70]: the first phase is to maximize the energy efficiency through an on–off power allocation method; and the spectrum efficiency is optimized in the second phase with a non-decreasing power allocation strategy. The authors of [71] introduced a directional glue-pouring algorithm to solve a similar problem. Due to the constant power consumption over time, a threshold of the transmission power in the directional glue-pouring algorithm needs to be calculated first, and then the process of glue-pouring is performed so that the power level is always higher than the threshold.

### 4.3. Discussions on Online Power Management

The offline power management is based on an assumption that the number and arriving time of energy/data packets in the future are pre-known [54], which is unrealistic. A viable solution to the online power management is to predict the future energy arrivals.

The prediction-based approach is based on two facts: (1) the time and the size of energy packets that will arrive in the near future are predictable to some extent; and (2) this prediction is sufficient to produce an online sub-optimal solution. As investigated in Section 2.2, the RF power density is quite stable over time in an outdoor environment. Therefore, the intensity of incident power is highly predictable, which makes the energy prediction feasible in the real world. Although the offline optimal transmission power is affected by the arrivals of all future energy packets, the energy packets to arrive in the near future has a much heavier impact on the power scheduling than the ones that will arrive in a far future. Therefore, if an energy harvester can predict the arrivals of energy packets in the near future, the performance of online power management can be close to the performance of the offline optimal solution.

*The statistics-based approach* will be a better fit in energy harvesting wireless systems when the RF power density in an indoor environment has considerable fluctuation due to human activity. It exploits the statistical characteristics of RF energy to develop online power management methods. In [72], the energy arrivals are modeled an independent and identically distributed Bernoulli process; the data transmission is modeled as MDP and each data has an associated importance value. The online transmission scheduling can be solved numerically by the policy iteration (PI), a dynamic programming algorithm. The device is scheduled to transmit the data if the importance value is above a dynamic threshold, which is a decreasing function of the battery’s energy level and discard the data otherwise. Blasco et al. [73] studied the situation when the transition probabilities of the Markov chain is unknown in the MDP model. In this work, the Q-learning algorithm is recommended to recursively estimate the expected discounted reward. It is shown that the performance of an online decision can be optimized after a reasonable learning period. Other similar online policies that use the MDP model for power management can refer to [74].

*The correlation-based approach* utilizes the correlation among energy packets for online power management. In [75], the energy harvesting process is modeled as a two-state Markov chain, where a good state represents that one energy unit is harvested in a time slot and a bad state means there is no energy received in that slot. If the length of each time slot is much shorter than the temporal variation of the RF energy, the energy strength between two neighboring time slots will become highly correlated. In the above model, the device decides to send or drop a data packet using the PI algorithm similar to [72,73]. A simplified suboptimal strategy, called the non-adaptive balanced policy (NABP), is available [75]. In NABP, the RF-EHWC devices compares the importance value of a data with a threshold to decide to send or to withdraw the current packet. The threshold is determined by the importance value of a data packet, the amount of harvested energy, and the residual energy in the battery. It aims to make the probability of data transmission equal to that of a good energy state in the current time slot.

## 5. Intermittent Connection Problem in RF-EHWC Networks

In the RF-EHWC networks, the energy harvesting module harvest energy from the ambient environment or from dedicated energy sources to provide sustainable energy for the nodes to monitor the environment or to communicate with other devices in the network. In this section, we discuss a unique challenge, called the intermittent connection problem, in RF-EHWC networks. The intermittent connectivity refers to the temporary connection loss resulted from nodes switching between “death” and “resurrection”.

Different from battery-powered devices that consume energy monotonically until the battery is exhausted, the energy level of RF-EHWC node could even rise after a data transmission due to newly harvested energy. Consequently, an RF-EHWC node will not “die” permanently but comes alive after energy cumulation. However, due to the thin RF energy in the environment, a small energy harvesting device usually consumes energy much faster than the energy harvesting rate [23]. Although it can resurrect with energy replenishment, an RF-EHWC node may become temporarily unavailable after continuous operation. This phenomenon is called the intermittent connections. The causes of intermittent connections and their impact on the routing protocol are analyzed in this section.

### 5.1. Cause of Intermittent Connections

The problem of intermittent connections is essentially caused by the slow energy harvesting rate of RF-EHWC system and the randomness of short-term traffic.

As summarized in Section 2.1, the power density of ambient RF energy is usually thin, which is in the order of nW/cm${}^{2}$ or even lower. With the limited power supply, an RF-EHWC device can only operate at an extremely low duty cycle (duty cycle is defined as the percentage of time that the transmitter can transmit using the energy received in a unit time). We use a low-power wireless communication module, Texas Instruments CC2500 (Texas Instruments, Dallas, TX, USA), as an example to offer an insight into how the low density of RF energy impacts the duty cycle of RF-EHWC node. Assume an energy harvester is equipped with a 2 dBi antenna receiving energy from the 900 MHz frequency band to power CC2500. Based on the effective aperture of this receiving antenna (i.e., 351 cm${}^{2}$ [76]) and highest energy density on 900 MHz frequency band (i.e., 1930 nW/cm${}^{2}$ [23]), the power provided by the harvester is 0.47 mW with 70% power conversion efficiency. When transmitting data at 1 mW (0 dBm), the power consumption of CC2500 is about 46.6 mW (21.2 mA × 2.2 V [77]), two orders higher than the energy harvesting rate. In this case, the duty cycle of CC2500 is at most 0.40%. This indicates that the RF energy harvested by an RF-EHWC device is far from enough for continuous data communications. Accordingly, energy harvesting nodes will become temporarily unavailable when the stored energy is depleted after intensive transmissions.

The randomness of short-term traffic is another reason that causes intermittent connections. It is common for nodes to have heterogeneous and highly dynamic traffic loads due to the nonuniform deployment of the network, dynamic channel quality, and variation of the surrounding environment. Depending on which routing protocol is running in the network, some special nodes, such as a cluster head in the hierarchical routing or a high-energy node in an energy-aware routing, may experience much heavier traffic than other nodes in a short term. This will not be a problem in conventional wireless sensor networks. The battery-powered nodes are resilient to the temporary variation of the local traffic since their lifetime is mainly determined by the long-term energy consumption. For this reason, the energy-aware routing proposed for the conventional wireless sensor network aims to balance the long-term traffic among nodes, whereas the short-term traffic unbalancing can be ignored [78]. In RF-EHWC networks, however, a burst of traffic can drain the harvested energy quickly and causes a node to be temporarily disconnected from the network. For a large-scale RF-EHWC network, the intermittent connections are very difficult to avoid since the short-term traffic is usually random and unpredictable [79]. Therefore, it is crucial to design routing protocols that can balance not only the long-term traffic but also the short-term traffic to mitigate the intermittent connections.

### 5.2. Impact of Intermittent Connections

The state of RF-EHWC nodes switching between alive and dead causes frequent and unpredictable changes of the network topology, which can pose a grand challenge on the resilient routing design. Although extensive routing protocols and topology management methods have been developed for wireless sensor networks [80], most of the existing protocols are vulnerable to the intermittent connections among nodes. How to maintain reliable communications among RF-EHWC nodes for packet delivery is a crucial problem.

#### 5.2.1. Frequent Changes of Network Topology

In real applications, the status of an RF-EHWC node may continuously switch between alive and dead, thereby causing frequent changes of the network topology. Compared with mobile ad hoc networks, the topology changes in an RF-EHWC network are faster and at a higher frequency due to the intermittent connection problem. Therefore, the routing protocols and topology management methods designed for mobile wireless networks may not work efficiently in RF-EHWC networks [81]. 

#### 5.2.2. Unpredictable Changes of Network Topology

To optimize the network performance in terms of the lifespan and throughput, a routing protocol needs to balance the energy consumptions among sensor nodes. In conventional wireless sensor networks, this can be achieved by carefully scheduling the sleep and active time of each individual according to its concurrent energy level [82], which results in highly controllable topology changes. In RF-EHWC network, however, the downtime of each node enforced by the energy deficiency is out of control in general. How long a node can stay active not only varies with the energy harvest rate but also depends on the temporary traffic load, which is barely predictable ahead of time.

To handle the above two challenges, the RF-EHWC network calls for the resilient routing protocol, which is capable of tackling the frequent and unpredictable disconnections among RF-EHWC nodes. It is also essential to eliminate or at least significantly reduce the overhead on restoring the network connectivity once the intermittent connections occur. 

## 6. Secure Communications

In this section, we introduce a unique security issue, which is mainly caused by the huge power difference between energy packets and data packets, in RF-EHWC. The potential methods to improve the communication security of RF-EHWC networks are also investigated.

### 6.1. Challenges in Secure Communication

In the physical layer, communication security is commonly enhanced by exploiting noises, interferences, or channel fading [83]. For instance, when the hybrid base station (HBS) serves as both data source and energy source, it can utilize an energy beam to create a strong co-channel interference to jam the communication channel of eavesdroppers [84]. In a multi-user network, the transmitting vector of HBSs can be optimized to maximize the secrecy rate, which is defined as the transmission rate that guarantees the perfect secrecy [85].

The power sensitivity of data communication and energy harvest modules have significant differences. In general, a data packet can be successfully decoded when the strength of the receiving signal is as low as −70 dBm (0.1 nW). By contrast, many energy harvesters need the minimum intensity of the incident power higher than −20 dBm (0.01 mW). It implies that HBS needs to consider the completely different power requirement of data and energy packets. It will be especially challenged when the HBS needs to deliver the energy and data information in a single packet to improve time efficiency. A high transmission power will increase the risk of information disclosure due to the broadcast nature of the wireless channel. Therefore, how to communicate securely while providing sufficient energy to RF-EHWC nodes is a critical problem. 

### 6.2. Artificial Noise Aided Method

The secrecy rate of wireless communications is determined by the difference in mutual information of the legitimate channel and eavesdropping channel. By adding the AN to the eavesdropping channel, the mutual information of the eavesdropping channel can be considerably reduced in defense of eavesdropping attack [86].

In the AN aided communication, based on a power-splitting coefficient, the transmission power is split into sending the confidential message and transmitting AN signal. The legitimate receiver removes the artificial interference from the received data via the noise cancellation based on the generation method of AN shared by the HBS. Through jointly optimizing the total transmission power and the power-splitting coefficient of the HBS, different objectives, such as a delay-limited secrecy information transmission or a no-delay-limited secrecy information transmission can be achieved. (For a delay-limited secrecy information transmission, the HBS aims to minimize the probability that the achievable secrecy rate at certain channel fading state below a specific threshold. For a no-delay-limited secrecy information transmission, the HBS aims at maximizing the ergodic secrecy capacity.)

**Figure 19:**
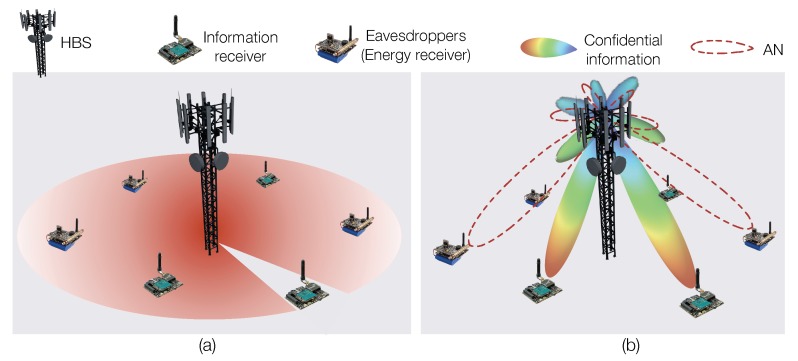
AN-aided secure communication in an RF-EHWC network: (**a**) without the knowledge of CSI; and (**b**) with the knowledge of CSI.

When the eavesdropper is an active transmitter, the HBS is able to measure the CSI of the eavesdropper. If the eavesdropper is passive but not completely silent, the HBS can also estimate the eavesdropping channel through measuring a local oscillator signal leaked from the front end of the eavesdropper’s circuitry [87]. The spatially selective AN can be performed to efficiently jam the information channel of the eavesdropper, as shown in Figure 19b. However, a perfect CSI is usually unavailable due to incorrect channel estimation, measurement quantization, and untimely channel feedback, especially in a large-scale RF-EHWC network [88]. As a result, many compromised transmission strategies are developed to balance the requirements between the CSI and the secrecy performance [89]. Consider a case where the eavesdropper is completely silent and thus no CSI of the eavesdropper can be perceived by the HBS. In this case, the HBS needs to jam eavesdroppers possibly at any possible location. The isotropic AN will be transmitted to make man-made noises be uniformly spread in the space except in the direction to the legitimate IR [90], as demonstrated in Figure 19a.

In [91], both perfect and imperfect CSI situations are investigated to optimize the secrecy rate. With perfect CSI, the secrecy rate maximization problem is non-convex and can be relaxed and reformulated into a series of convex subproblems by using the semi-definite relaxation (SDR) and successive convex approximation methods. If the CSI is imperfect, the AN-aided transmission vector can be designed based on the worst-case model. The objective becomes maximizing the secrecy rate under any channel estimation error in the uncertainty region. To reach the best performance, the optimization problem is first converted into a quadratic matrix inequality problem. Afterward, by converting the constraints of the semi-infinite quadratic matrix inequality into that of the linear matrix inequality using the S-procedure [92] and the Schur complement [93], solvable semidefinite programs can be eventually constructed. In [94], the AN transmission combined with the cognitive radio and the non-orthogonal multiple access (NOMA) technologies in a multi-user network is studied. 

## 7. Potential Applications

The energy harvesting technology is penetrating into many hot areas, such as the IoT, BAN, and 5G communications, providing realistic solutions for large-scale, sustainable, and low-cost environment monitoring and wireless information exchange system. In this section, we present several existing and potential applications of energy harvesting wireless communications. 

### 7.1. Healthcare of Animals

Studies have revealed that animals usually hide their illness and pretend to be good even when they are sick. This feature makes it difficult for veterinarians to save animal’s lives once clear illness symptoms appear. Therefore, how to monitor the health status of a large number of animals in a zoo or on a farm becomes an important but challenging issue.

**Figure 20:**
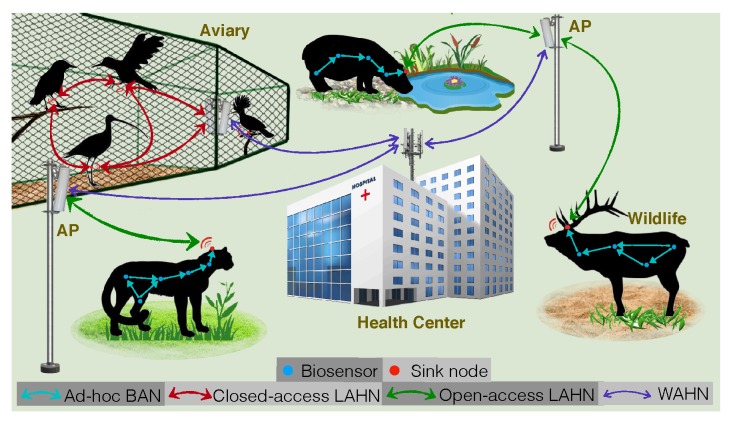
A 3-tier architecture of an RF-EHWC network for the healthcare of animals [95].

The multi-tier RF-EHWC network provides a cost-effective solution, as shown in Figure 20. In Tier 1, energy harvesting biosensors are implanted into an animal’s body and form an ad hoc BAN. In Tier 2, each BAN is associated with a sink node to form a local area healthcare network (LAHN). The sink node is embedded in a collar, an ear tag, or a leg band of an animal. It is responsible for forwarding the data collected by the biosensors to the nearest AP. In Tier 3, a wide area healthcare network is established by connecting all APs into Ethernet for real-time health monitoring.

### 7.2. Wearable Devices

In recent years, wearable devices, such as the smart watch and the smart bracelet, have become very popular for health monitoring and physical activity tracking. Commercial wearable devices are often designed as a stand-alone ecosystem that integrates the information collection, data processing, wireless communication, and message display in a single device. The high energy consumption makes current wearable electronics heavily depend on high capacity batteries.

If we separate the data processing and information display modules and integrate only sensing and data transmission into wearable devices, the energy consumption of the wearable devices will be greatly reduced so that they can be powered by renewable energy sources. In [96], a researcher conducted an experiment to charge a wearable devices with a dedicated energy source held in the right hand. The transmission power and the frequency band of the energy source were 2 W and 464–468 MHz, respectively. According to the experiment results, the device attached on the back received the lowest power, which was −12 dBm on average; the wearable device attached on the front of the head can harvest the highest power, which can reach an average of 6.9 dBm.

To obtain more energy for the reliable operation, the wearable device can choose a hybrid energy harvester. In [97], the recommended energy sources are the thermal energy and RF energy. A small body-worn thermoelectric generator (TEG) collects thermal energy from the human body and typically provides output voltages lower than <100 mV. It requires an ultra-low voltage multiplier to boost the low output voltage from <100 mV to 1.35 V. However, the applied voltage multiplier requires a 600 mV voltage for startup. Therefore, an RF rectifier of −10 dBm sensitivity is used to harvest radio energy, and provide the required startup voltage. The implementation of other energy harvesters for wearable devices can refer to [98]. 

### 7.3. 5G-Assisted RF-EHWC

As investigated in Section 2.1, the power density of ambient RF energy is low due to the high spreading loss of EM waves in the free space and the safety concerns. The 5G mobile technique can alleviate this low power density problem and provide an opportunity for RF-EHWC networks to harvest energy in a more efficient and flexible manner. 

**Figure 21:**
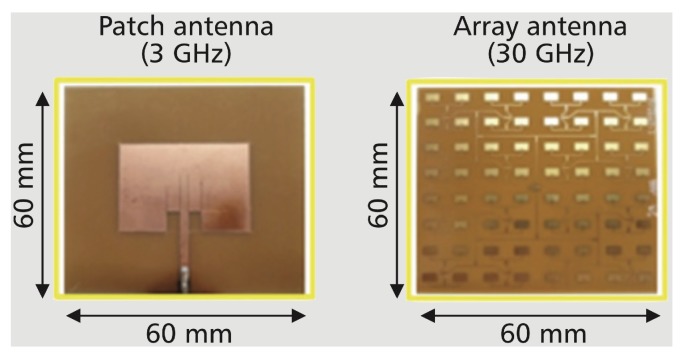
Antenna comparisons [105]: Patch antenna for traditional wireless communications in 3 GHz; Antenna array with 64 (8×8) elements for 5G communications in 30 GHz.

By increasing the communication frequency to the mmWave band, the size of the antenna can be greatly reduced, as illustrated in Figure 21. This enables dense deployment of small femtocells with a large number of antennas in a 5G network [99]. The high-density deployment of the antenna array is beneficial to the application of an RF-EHWC system from the following three perspectives:

- *Large antenna gain*: Using an array antenna, HBS (femtocells) can create a narrow beam to increase the antenna gain for efficient power transmission or enhance the spatial multiplexing gain for high-speed data transmission. As tested in [100], if both sender and receiver are equipped with the array antenna that is demonstrated on Figure 21, the strength of RF waves arrived at the receiver can be 20 dB higher than the situation with a single antenna.
- *High beam-steering resolution*: The mmWave phased array can precisely form a large number of controllable beams. With a 32-element phased array, the difference between the directions of neighboring beams can be as low as 1.4 degrees [101]. The high resolution of the beam-steering offers the HBS a high degree of freedom to allocate power for information and energy transmissions. It is even possible to supply power and send data to adjacent RF-EHWC devices simultaneously. At the receiver side, the RF-EHWC device with massive antennas can harvest energy and receive information from different HBSs at the same time via the antenna-switching RF-EHWC scheme [102,103].
- *Low propagation loss*: Ultra-dense networking is considered a promising technique for 5G communications [104]. The high-density deployment of the HBS can shorten the distance between the energy source and RF-EHWC devices. Therefore, a low propagation loss of the RF energy can be expected in a 5G-assisted RF-EHWC network.

## 8. Conclusions

RF-EHWC is an attractive technology to provide large-scale wireless sensor networks green, stable, and self-sustainable power supply. In this article, we have presented a comprehensive survey of the latest developments in the broad field of RF-EHWC technology. The survey covered a variety of topics including the ambient RF environment, the circuit design of an RF-EHWC system, and practical issues in the emerging paradigm of power management, routing design and security in RF-EHWC networks. Some potential applications of the RF-EHWC technology have also been introduced. In order to maximize network performance, the practical issues, such as nonlinearity of harvester circuit, nonlinearity of battery charging, battery imperfection, thin RF energy, and intermittent connection problem, need to be considered in the real system design. We hope that this survey can help move researches forward along this road.

## Figures and Tables

**Table 1:** The power density of different energy sources.

Source	Conditions		Density
Solar [37]	Morning	Unshaded	20 mW/cm${}^{2}$
		Shaded by tree	3 mW/cm${}^{2}$
	Noon	Unshaded	60 mW/cm${}^{2}$
		Shaded by tree	10 mW/cm${}^{2}$
RF (Average) [23]	DTV	470–610 MHz	0.89 nW/cm${}^{2}$
	GSM900 (MT) ^†^	880–915 MHz	0.45 nW/cm${}^{2}$
	GSM900 (BT) ^‡^	920–960 MHz	36 nW/cm${}^{2}$
	GSM1800 (MT) ^†^	1710–1785 MHz	0.5 nW/cm${}^{2}$
	GSM1800 (BT) ^‡^	1805–1880 MHz	84 nW/cm${}^{2}$
	3G (MT) ^†^	1710–1785 MHz	0.46 nW/cm${}^{2}$
	3G (BT) ^‡^	2110–2170 MHz	12 nW/cm${}^{2}$
	WiFi	2.4–2.5 GHz	0.18 nW/cm${}^{2}$
Thermal [38]	Human body (4×8 cm)	10 K temp diff	0.22 nW/cm${}^{2}$
		35 K temp diff	0.47 nW/cm${}^{2}$
Piezoelectric	Push bottom [39]		2 mJ/N
	Human biomechanics [40]		7.34 μW/cm${}^{3}$

^‡^ BT, Base transmitter. ^†^ MT, Associated mobile transmitter.
